# *In Vivo* Regulation of E2F1 by Polycomb Group Genes in *Drosophila*

**DOI:** 10.1534/g3.112.004333

**Published:** 2012-12-01

**Authors:** Jun-Yuan Ji, Wayne O. Miles, Michael Korenjak, Yani Zheng, Nicholas J. Dyson

**Affiliations:** * Department of Molecular and Cellular Medicine, College of Medicine, Texas A&M Health Science Center, College Station, Texas 77843-1114; † Massachusetts General Hospital Cancer Center, Laboratory of Molecular Oncology, and Department of Pathology, Harvard Medical School, Charlestown, Massachusetts 02129

**Keywords:** cell proliferation, E2F1, Su(z)2, PcG, *Drosophila*

## Abstract

The E2F transcription factors are important regulators of the cell cycle whose function is commonly misregulated in cancer. To identify novel regulators of E2F1 activity *in vivo*, we used *Drosophila* to conduct genetic screens. For this, we generated transgenic lines that allow the tissue-specific depletion of dE2F1 by RNAi. Expression of these transgenes using Gal4 drivers in the eyes and wings generated reliable and modifiable phenotypes. We then conducted genetic screens testing the capacity of Exelixis deficiencies to modify these E2F1-RNAi phenotypes. From these screens, we identified mutant alleles of *Suppressor of zeste 2* [*Su(z)2*] and multiple Polycomb group genes as strong suppressors of the E2F1-RNA interference phenotypes. In validation of our genetic data, we find that depleting Su(z)2 in cultured *Drosophila* cells restores the cell-proliferation defects caused by reduction of dE2F1 by elevating the level of *dE2f1*. Furthermore, analyses of methylation status of histone H3 lysine 27 (H3K27me) from the published modENCODE data sets suggest that the genomic regions harboring *dE2f1* gene and certain *dE2f1* target genes display H3K27me during development and in several *Drosophila* cell lines. These *in vivo* observations suggest that the Polycomb group may regulate cell proliferation by repressing the transcription of *dE2f1* and certain dE2F1 target genes. This mechanism may play an important role in coordinating cellular differentiation and proliferation during *Drosophila* development.

The E2F family of transcription factors provides temporal control of genes that are necessary for the G1/S-phase transition and are critical for controlling cell proliferation (Burkhart and Sage 2008; van den Heuvel and Dyson 2008). In early G1 phase of the cell cycle, the RB family proteins bind to and inhibit E2F transcriptional activities. In late G1 to S phase, cyclin-dependent kinases (CDKs) phosphorylate the RB family proteins, which then dissociate, resulting in E2F liberation and activation of E2F-dependent transcription (Burkhart and Sage 2008; van den Heuvel and Dyson 2008). E2F-regulated genes are required in dividing cells for proper DNA replication and subsequent mitosis (Müller and Helin 2000; Ren *et al.* 2002). The basic unit of E2F is a heterodimer composed of an E2F and a DP subunit. Eight *E2F* genes have been characterized in mammals (Stevaux and Dyson 2002; Trimarchi and Lees 2002; van den Heuvel and Dyson 2008): three activating E2Fs (E2F1∼3), two DP interacting repressive E2Fs (E2F4∼5), and three DP independent repressive E2Fs (E2F6∼8). The E2F family members display partial redundancy as well as antagonizing functions; thus, it is challenging to elucidate the functions of individual mammalian E2Fs. The RB-E2F pathway is streamlined in *Drosophila* because it contains only two E2Fs, the activator dE2F1 and the repressor dE2F2 (Frolov and Dyson 2004). Therefore, genetic and developmental analyses using *Drosophila* as a model organism may provide important insights into the mechanisms regulating the RB-E2F pathway during development.

We used a modifiable dE2F1 RNA interference system in *Drosophila* to identify novel regulators of E2F1 activity. By conducting a dominant modifier genetic screen, we have identified a set of genetic interactions between dE2F1 and members of the Polycomb group (PcG) genes. Several PcG complexes have been characterized, including polycomb repressive complex 1 (PRC1), PRC2, *Pho*-repressive complex (PhoRC), dRING-associated factors, and the Polycomb repressive deubiquitinase complex [PR-DUB (Levine *et al.* 2004; Schuettengruber *et al.* 2007; Schwartz and Pirrotta 2007; Müller and Verrijzer 2009; Margueron and Reinberg 2011)]. Of these complexes, the PRC2 contains the sole histone methyl-transferase, Enhancer of zeste (E(z)), specific for histone 3 lysine 27 (H3K27). Methylation of H3K27 by PRC2 is shown to facilitate the recruitment of the PRC1 complex through direct binding with the chromodomain of Polycomb (Pc) (Cao *et al.* 2002; Fischle *et al.* 2003; Min *et al.* 2003; Cao and Zhang 2004). However, *in vivo* regulations of these complexes in development are less well understood.

There are several reports linking PcG complexes to the RB-E2F pathway in vertebrates. First, the *INK4b-ARF-INK4a* tumor suppressor locus is regulated by the PcG complexes (Gil and Peters 2006). The *INK4b-ARF-INK4a* locus is vertebrate-specific and encodes the INK4 family of inhibitors that target CDK4/6-cyclin D (CycD), which phosphorylate and inactivate pRB family members in mammals (Sherr 2004; Gil and Peters 2006). Second, RB was reported to regulate the G2/M-phase transition by forming an E2F-RB-CtBP-HPC2 complex, thus repressing the expression of cyclin A and Cdc2 in cultured human cells (Dahiya *et al.* 2001). Third, E2F6, one of the repressive E2F family members in mammals, forms complexes with RYBP, Bmi1, EPC1, and other PcG subunits (Trimarchi *et al.* 2001; Ogawa *et al.* 2002; Attwooll *et al.* 2005) and regulates Hox gene expression and axial skeleton development in mouse (Storre *et al.* 2002; Courel *et al.* 2008). Finally, the RB-E2F pathway has been shown to regulate the expression of certain PcG subunits, such as EZH2 and EED (Bracken *et al.* 2003). Although it is not known whether these mechanisms are conserved in evolution, these studies suggest that the interactions between the RB-E2F pathway and PcG-mediated silencing can occur at multiple levels.

In *Drosophila*, PcG complexes have been reported to regulate the expression of several cell-cycle regulators. Polycomb responsive elements have been identified in the promoter and coding region of *dCycA* and *dE2f1* (Martinez *et al.* 2006). Similarly, the PhoRC subunit Pleiohomeotic (Pho) and the PRC1 component Ph are found at the promoters of *dCycB*, *dDp*, *dE2f1*, and *Rbf1* in *Drosophila* embryos (Oktaba *et al.* 2008). These studies suggest a direct role for multiple PcG complexes in regulating key Rb-E2F pathway components and that PcG complexes may affect cell proliferation by controlling the expression of different cell-cycle regulators in development. The relationships between PcG complexes and cell proliferation in different developmental contexts are important and far from clear, thus further investigations using diverse model systems and approaches are necessary.

We have identified a set of genetic interactions between PcG genes and dE2F1. As summarized in this report, our results suggest that PcG complexes may directly repress the transcription of *dE2f1* and certain dE2F1 target genes. Together with the previous reports linking PcG complexes to cell-cycle regulators (Martinez *et al.* 2006; Oktaba *et al.* 2008), our genetic analyses provide *in vivo* evidence that supports a role for different PcG complexes in coordinating cell proliferation and differentiation during *Drosophila* development by controlling the expression of several key cell-cycle regulators.

## Materials and Methods

### Generation of *UAS-dE2f1-dsRNA* (tissue-specific *dE2f1*-RNAi) transgenic lines

A 650-bp fragment of DNA sequence was amplified by polymerase chain reaction (PCR) using *dE2f1* cDNA as the template, and the primer sequences were 5′-TTATTTCAAACGCCCTACCG-3′ and 5′-GAATTGCATCTGCAGTGAGC-3′. This fragment was previously used as the target sequence to generate double-strand RNA (dsRNA) in our microarray analyses for dE2F1 target genes (Dimova *et al.* 2003). The PCR product was gel purified and subsequently subcloned into the pWIZ vector in an inverted configuration [for the detailed procedure, see (Lee and Carthew 2003)] and verified by sequencing. The final *pWIZ-dE2f1-dsRNA* vector, as referred to as “*UAS-dE2f1-dsRNA*” in the text, was injected into early *Drosophila* embryos (*w^1118^*) to generate transgenic flies. Approximately 30 different transgenic lines carrying one or multiple transgenes, as indicated by their eye color because pWIZ carries mini-*white* as a selection marker, were balanced, crossed, and recombined with different Gal4 lines using standard genetic crosses. Because the *dE2f1dsRNA* phenotypes in both the eye (*w^1118^*; *GMR-Gal4*, *UAS-dE2f1RNAi #10* or *#8/+*; *+/+* at 25°) and the wing (*w^1118^*; *ptc-Gal4*, *UASdE2f1dsRNA#3/+*; *+/+* at 22∼23°) are modifiable by known RB-E2F pathway factors in expected manners and the phenotypes are fully penetrate, these two recombined stocks were used for genetic analyses in this work.

### Genetic screen using the Exelixis deficiency (*Df*) lines

Flies were maintained on standard cornmeal-yeast agar medium. Exelixis *Df* lines and most of the mutant alleles used in this work were obtained from the Bloomington *Drosophila* Stock Center. The null allele of *Polycomb* (*Pc^3^*) allele was obtained from Dr. Antonio Garcia-Bellido (Castelli-Gair *et al.* 1990). For genetic screen using the Exelixis *Df* lines: approximately 5∼10 female virgins from either *w^1118^*; *GMR-Gal4*, *UAS-dE2f1RNAi #10* (or *#8*)/*CyO*; *+/+*, or *w^1118^*; *ptc-Gal4*, *UASdE2f1dsRNA#3/CyO*; *+/+* lines were crossed with 5∼10 males from each *Df* line on second or third chromosomes, and the crosses were maintained at either 25° (for the eye phenotype) or 22∼23° (for the wing phenotype). As an example for the eye phenotype, the female F1 with the following genotypes were scored for potential modifications: *w^1118^*; *GMR-Gal4*, *UAS-dE2f1RNAi #10/Df(2R/2L)Exel#*; *+*, or *w^1118^*; *GMR-Gal4*, *UAS-dE2f1RNAi #10/+*; *Df(3R/3L)Exel#/+*. The reverse crosses were performed for *Df* lines on the X chromosome and F1 female flies with the following genotype were scored: *Df(1)Exel#/w^1118^*; *GMR-Gal4*, *UAS-dE2f1RNAi #10/+*; *+/+*.

### Scanning electron microscopy and measurement of the L3-L4 intervein region

The F1 female flies were stepwise dehydrated using ethanol, and scanning electron micrographs were taken following standard procedures at the Northeastern University. To measure L3-L4 intervein region, wings are removed, briefly treated with isopropanol and then mounted in Canada Balsam (Sigma-Aldrich, St. Louis, MO). The width of L3-L4 was measured under a Nikon i90 microscope using the Nikon NIS Elements software.

### *Drosophila* RNAi in SL2 cells and the MTT assay

The dsRNAs used in this work were synthesized using the RiboMax Large Scale RNA Production Systems (Promega, Madison, WI) following the manufacturer’s instructions. The following primer sets were used to generate dsRNAs to *dE2f1* (F: 5′-CGAGTAAGAAGCAGCAGCAC; R: 5′-CTGCCGGTTCTATCGTGATT), *Su(z)2* (F: 5′-TCTGCTACCGGATTCTGCTTTACG; R: 5′-AACTCCCTTTCGATTCGCTGTCTT), *Psc* (F: 5′-CAACGCCAAGCCGAACATCAAATC; R: 5′-AGCGGCTGGGGCGACTCATAAAC), *Pc* (F: 5′-TGCCAATGCAATAGATTGTAAA; R: 5′-CGCTTTGAATTGCTGTTTTG), *E(Pc)* (F: 5′-TCAGCCCTTCTACGATGCCTACTA; R: 5′-CTCGCGTCGCCTCACCATCTCCAG), and *white* with T7 sequence (F: 5′-CTAATACGACTCACTATAGGGAGGGAAGATGGCTCCG; R: 5′-CTAATACGACTCACTATAGGGAGTTTCGCTCAGCAAATG). Treatment of the *Drosophila* SL2 cells with 50 μg of dsRNA was performed as described previously (Dimova *et al.* 2003). The *white*-dsRNA was used as a control, and it is also used to normalize the total amount of dsRNA in codepletion experiments. The MTT assay was performed as described (Hansen *et al.* 1989) in 96-well format, and the O.D. at 570nm was measured using a standard plate-reader.

### RNA preparation and quantitative reverse-transcription (qRT)-PCR analysis

The total RNA isolation, quantification, reverse transcription, and the subsequent qRT-PCR analyses were performed as described previously (Zhao *et al.* 2012). The following primers were used for qRT-PCR for data presented in Figure 4: *stg* (F: 5′-AAACCAGCTGCTCGGCATATT; R: 5′-ATCTCAATTCACCGAACGAGGA), *rnrL* (F-5′-CGGTTAAGGCTCAATCCCTGT; R: 5′-TGGTTGCTCTTCCTGTTGCA), *his2AvD* (F: 5′-TCACTCCTCGCCACTTACAGCT; R: 5′-CGACTTGTGTATGTGCGGAATG), *Mars* (F: 5′-ATCTTGGATCCTCAGCAGACGA; R: 5′-GGCATTCCATTGGATTCGC), *Mcm 5* (F: 5′-GAAGCTAAAGAGCCGCTACGTG; R: 5′-TCCAACTGACGCACAGTGATG), *PCNA* (F: 5′-GAATCGGCTAACCAGGAGAAGG; R: 5′-ACCACGCACGAGAAGTCTGTCT), *Nebbish* (F: 5′-AGTCGCATTGCCCTTAATCTGA; R: 5′-ATGTCTGTCGCGGTGTATTGC), *dE2f1* (F: 5′-CTCTTTCTCCGCGTGTGGATT; R: 5′-GCGACGAAAAGCGAACTGAA), *dCycA* (F: 5′-AACCACGAACCGCTGAACAA; R: 5′-GGCAGCGTTGGAATTAGTTT), *dCycE* (F: 5′-ATGTGGCGCATAAGGTGCA; R: 5′-CCCGATCTTTGGCGGATAA), and *rp49* gene (F: 5′-ACAGGCCCAAGATCGTGAAGA; R: 5′-CGCACTCTGTTGTCGATACCCT) was used as the internal loading control.

## Results

### Tissue-specific knockdown of dE2F1 activity produces modifiable phenotypes

**Figure 1:**
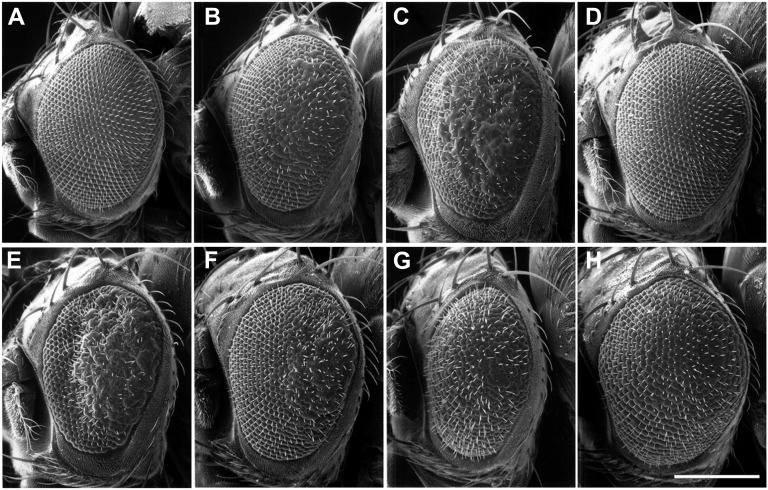
Tissue-specific expression of dE2f1-dsRNA generates phenotypes that can be modified by known factors of the dE2F1 pathway. (A) A normal *Drosophila* eye (*w^1118^*; *GMR-Gal4/*+; +/+). (B) Expressing one copy of the *UAS-dE2f1-dsRNA* (Line #10) generates a slight rough eye phenotype (*w^1118^*; *GMR-Gal4*, *UAS-dE2f1dsRNA#10/*+; +/+), which can be enhanced by reducing the endogenous *dE2f1* levels, as shown in (C) (*w^1118^*; *GMR-Gal4*, *UAS-dE2f1dsRNA#10/*+; *dE2f1^i2^/*+), and completely rescued by overexpressing wild-type *dE2f1*, as shown in (D) (*w^1118^*; *GMR-Gal4*, *UAS-dE2f1dsRNA#10/*+; *UAS-dE2f1*^+^/+). A stronger rough eye phenotype is generate when multiple copies of *UAS-dE2f1dsRNA* (line #8) is expressed, as shown in (E) (*w^1118^*; *GMR-Gal4*, *UAS-dE2f1dsRNA#8/*+; +/+). This stronger phenotype can be suppressed by overexpressing wild-type *dCycA* (F: *w^1118^*; *GMR-Gal4*, *UAS-dE2f1dsRNA#8/*+; *UAS-dCycA*^+^/+), wild-type dCycE (G: *w^1118^*; *GMR-Gal4*, *UAS-dE2f1dsRNA#8/UAS-dCycE*^+^; +/+), or *dCdk4* and *dCycD* (H: *w^1118^*; *GMR-Gal4*, *UAS-dE2f1dsRNA#8/*+; *UAS-dCdk4*^+^, *UAS-dCycD*^+^/+). The scale bar (in H) is 200µm.

Homozygous *dE2f1* mutant animals die during larval development (Duronio *et al.* 1995); thus, we used a *dE2f1-dsRNA* expression system based on the pWIZ vector (Lee and Carthew 2003). This system allows the tissue-specific expression of the target dsRNA (Hannon 2002) using the Gal4-UAS system (Brand *et al.* 1994; Lee and Carthew 2003). We generated multiple transgenic lines that produce a 650-bp dsRNA from the *dE2f1* gene under control of the UAS, designated as “*UAS-dE2f1-dsRNA*” (see *Materials and Methods* for details). The *UAS-dE2f1-dsRNA* transgenes were then crossed to multiple tissue-specific Gal4 drivers and the resulting phenotypes were characterized. By driving the expression of *UAS-dE2f1-dsRNA* using the eye-specific *GMR-Gal4* and the wing-specific *patched-Gal4* (*ptc-Gal4*), we observed phenotypes with 100% penetrance and limited variation. Expression of *dE2f1-dsRNA* under the control of GMR-Gal4 caused a rough eye phenotype characterized by fused ommatidia (Figure 1B, compared with the control in Figure 1A), which we refer to as the “*dE2f1-dsRNA* eye phenotype” hereafter. Expression of *dE2f1-dsRNA* under the control of *ptc-Gal4* reduces the L3-L4 intervein region in the adult wing (Figure 3B, compared with the control in Figure 3A), which is referred as the “*dE2f1-dsRNA* wing phenotype.”

To verify the specificity of the *dE2f1-dsRNA*−induced phenotypes, we recombined different *UAS-dE2f1-dsRNA* lines to the *GMR-Gal4* or *ptc-Gal4* drivers on the second chromosome (see *Materials and Methods*). Using these stocks, we then tested the capacity of components of the Rb-E2F pathway to modifying the phenotypes. We observed that the *GMR*-driven rough eye phenotypes generated by a weak allele of *dE2f1-dsRNA* (line #10; Figure 1B) were enhanced by mutant alleles of *dE2f1* (Figure 1C). In contrast, the rough-eye phenotypes can be suppressed by introducing a single copy of a *UAS-dE2f1^+^* transgene (Figure 1D). We observed that even the strong effects of *dE2f1-dsRNA* (line #8; Figure 1E) were suppressed by the overexpression of wild-type dCycA (Figure 1F), dCycE (Figure 1G), or dCdk4-dCycD (Figure 1H). Conversely, mutant alleles of *dCdk4*, *dCycA*, or *dCycE* enhanced the *dE2f1-dsRNA* phenotypes (data not shown). These genetic analyses show that the *dE2f1-dsRNA* phenotypes are modified by components of the Rb-E2F pathway in a predictable manner, suggesting that the phenotypes are caused by specific reduction of dE2F1 activity. In support of this, we observed reduced dE2F1 protein levels in both immunostaining and Western blotting experiments when using tissue-specific expression of *dE2f1*-dsRNA (Morris *et al.* 2008). We also find that knockdown of dE2F1 in the wing imaginal discs results in reduced expression of a PCNA-GFP reporter, which directly reflects endogenous dE2F1 activity (Thacker *et al.* 2003; Morris *et al.* 2008). Taken together, these molecular and genetic analyses suggest that the *dE2f1-dsRNA* phenotypes result from the specific reduction of dE2F1 activity.

### A dominant modifier genetic screen to identify novel regulators of dE2F1 activity

**Figure 2:**
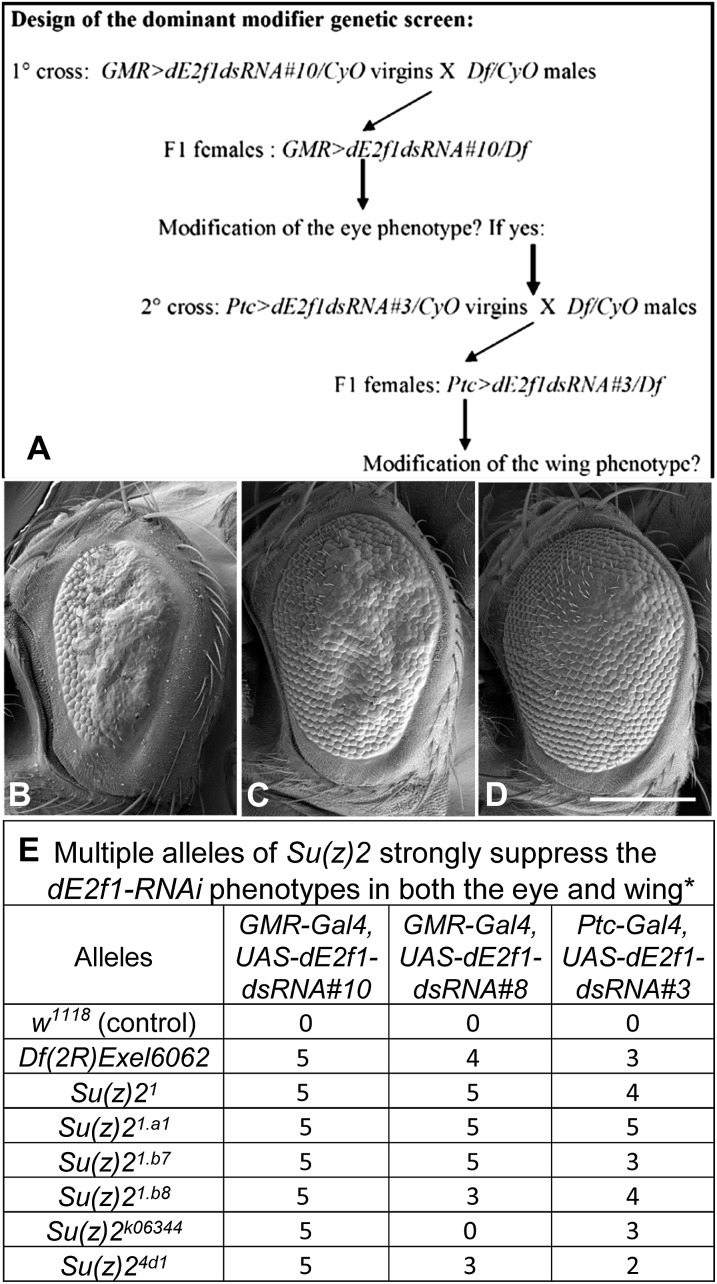
*Su(z)2* is a strong suppressor of the *dE2f1-dsRNA* phenotypes in the eye. (A) The design of the dominant modifier genetic screen using deficiency lines. (B-D) shows the modification of the *dE2f1-dsRNA* eye phenotype by *Su(z)2* alleles. The eye phenotype of *GMR-Gal4*, *UAS-dE2f1dsRNA#8/*+ (B) flies can be strongly suppressed by the *Df(2R)Exel6062* line (C, the genotype is *w^1118^*; *GMR-Gal4*, *UAS-dE2f1dsRNA#8/Df(2R)Exel6062*; +/+) and a null allele of *Su(z)2* (D, the genotype is *w^1118^*; *GMR-Gal4*, *UAS-dE2f1dsRNA#8/Su(z)2^1.b7^*; +/+). (E) Summary of the genetic interactions between *Su(z)2* alleles and *dE2f1-dsRNA* phenotypes in the eye and wing. The suppressive effect was ranked with scores from 1 to 5, with “1” being the weakest and “5” the strongest. “0” means no genetic interaction. The scale bar in (D) is 200µm.

To identify novel regulators of dE2F1 *in vivo*, we performed a dominant modifier genetic screen based on the *dE2f1-dsRNA* phenotypes described previously. The initial screen used the Exelixis *Df* collection (459 lines), which was generated in an isogenic background and all of the breakpoints are molecularly defined (Parks *et al.* 2004). We conducted a primary screen using the *dE2f1-dsRNA* eye phenotype because of ease of screening, and only *Df* lines that were able to modify this eye phenotype were subsequently retested using the *dE2f1-dsRNA* wing phenotype (Figure 2A). Thus, the *Df* lines that did not modify the *dE2f1-dsRNA* eye phenotype (referred to as “no effect” or “NE” in the tables) are excluded from further analysis (referred to as “not determined” or “ND” in the tables). Although this screen strategy may miss the modifiers that only affect the *dE2f1-dsRNA* wing phenotype, it enabled the identification of general regulators of E2F1 activity rather than tissue-specific modifiers.

From these screens, we identified 18 suppressor *Df* lines (Table 1) and 23 enhancer *Df* lines (Table 2) that modified both *dE2f1-dsRNA* phenotypes in the same fashion. The results of all Exelixis *Df* lines are summarized in Supporting Information, Table S1. Because the *dE2f1-dsRNA* phenotypes are based on RNAi, we tested the modifier *Df* lines on the *GMR > white-Inverted Repeat* (*GMR-w^IR^*) line, to identify gene products that change RNAi efficiency rather than the E2F1 directly (Lee *et al.* 2004). None of the enhancers and suppressors of the *dE2f1-dsRNA* phenotypes affected the *GMR-w^IR^* eye color (Table 1 and Table 2), suggesting that the modifiers identified in our screen are *bona fide* regulators of dE2F1.

**Table 1:** Exelixis *Df* lines that dominantly suppress the *dE2f1-dsRNA* phenotypes

Bloomington Stock No.	Symbol	Breakpoints	*GMR-Gal4,UAS-dE2f1-dsRNA#10**^a^*^,^*^b^*	*ptc-Gal4,UAS-dE2f1-dsRNA#3**^a^*^,^*^c^*	*GMR-w^IR^* *^a^*
7699	Df(1)Exel6221	1B4;1B8	5	2	NE
7700	Df(1)Exel6223	1C4;1D2	5	4	NE
7723	Df(1)Exel6255	20A1;20B1	5	3	NE
7772	Df(2L)Exel7002	21B4;21B7	5	2	NE
7774	Df(2L)Exel8003	21D1;21D2	5	2	NE
7489	Df(2L)Exel6002	21D2;21D3	5	5	NE
8000	Df(2L)Exel6006	22B5;22D1	5	2	NE
7817	Df(2L)Exel8024	31A2;31B1	5	2	ND
7531	Df(2L)Exel6049	40A5;40D3	5	1	NE
7540	Df(2R)Exel6058	44C4;44D1	5	2	NE
7544	Df(2R)Exel6062	49E6;49F1	5	4	NE
7880	Df(2R)Exel9015	51F11;51F12	5	1	NE
7883	Df(2R)Exel7138	52D1;52D12	5	1	NE
7557	Df(2R)Exel6077	57F10;58A3	5	4	NE
7903	Df(2R)Exel7173	58D4;58E5	5	2	NE
7921	Df(3L)Exel9000	64A10;64B1	5	2	NE
7927	Df(3L)Exel7210	65A1;65A5	5	2	NE
7992	Df(3R)Exel9014	95B1;95D1	5	1	NE

a The suppressive effect was ranked with scores from 1 to 5, with “1” the weakest and “5” the strongest. ND, not determined (this line is no longer available from the Bloomington stock center); NE, no effect.

b These crosses were maintained at 25°.

c These crosses were maintained at 22-23°; see *Materials and Methods* for the detailed genotypes analyzed.

**Table 2:** Twenty-three enhancers from the Exelixis Df lines

Bloomington Stock No.	Symbol	Breakpoints	*GMR-Gal4,UAS-dE2f1- dsRNA#10**^a^*^,^*^b^*	*ptc-Gal4,UAS-dE2f1- dsRNA#3**^a^*^,^*^c^*	*GMR-w^IR^* *^a^*
7510	Df(2L)Exel6027	32D2;32D5	5	Lethal	NE
7519	Df(2L)Exel6036	35B1;35B2	3	5	NE
7859	Df(2R)Exel7094	44A4;44B4	3	4	NE
7538	Df(2R)Exel6056	44A4;44C2	4	5	NE
7896	Df(2R)Exel7162	56F11;56F16	2	3	NE
7554	Df(2R)Exel6072	57B16;57D4	2	Lethal	NE
7902	Df(2R)Exel7171	58C1;58D2	5	Lethal	NE
7745	Df(3L)Exel6279	66A17;66B5	4	2	NE
7602	Df(3L)Exel6123	70D7;70E4	Pupal lethal	Lethal	NE
7611	Df(3L)Exel6132	74B2;74D2	3	1	NE
7614	Df(3L)Exel6135	76B11;76C4	5	Lethal	NE
7624	Df(3R)Exel6145	83C1;83C4	5	Lethal	NE
7627	Df(3R)Exel6148	84F12;85A2	Pupal lethal	Lethal	NE
7632	Df(3R)Exel6153	85D21;85E1	3	3	NE
7633	Df(3R)Exel6154	85E9;85F1	4	2	NE
7732	Df(3R)Exel6265	85F10;85F16	4	2	NE
7636	Df(3R)Exel6157	86B1;86B3	5	2	NE
7641	Df(3R)Exel6162	87A1;87B5	Pupal lethal	Lethal	NE
7649	Df(3R)Exel6170	87F10;87F14	1	5	NE
7742	Df(3R)Exel6275	88D1;88D7	5	Pupal lethal	NE
7659	Df(3R)Exel6180	91B5;91C5	3	3	NE
7678	Df(3R)Exel6199	95F8;96A2	3	5	NE
7993	Df(3R)Exel8178	95F8;96A6	3	4	NE

a The effect of enhancement was ranked with scores from 1 to 5, with “1” the weakest and “5” the strongest. NE, no effect.

b These crosses were maintained at 25°.

c These crosses were maintained at 22-23°; See *Materials and Methods* for the detailed genotypes analyzed.

### *Su(z)2* is a strong suppressor of the *dE2f1-dsRNA* phenotypes

**Figure 3:**
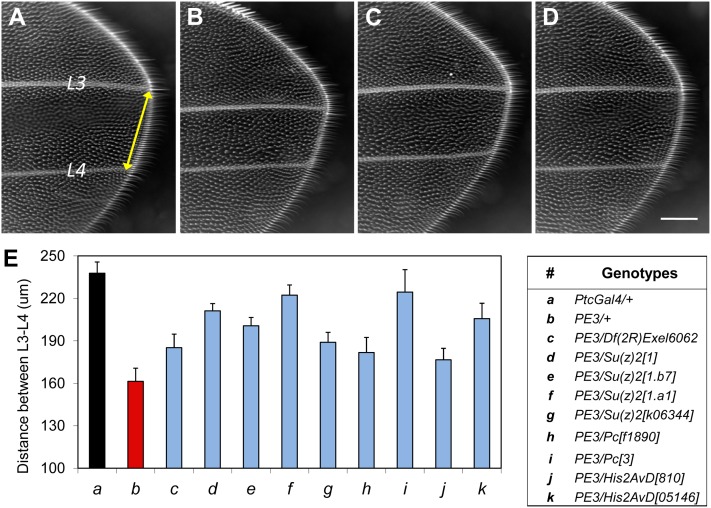
*Su(z)2* and additional PcG genes are strong suppressors of the *dE2f1-dsRNA* phenotypes in the wing. (A) Part of L3-L4 intervein region of a control *Drosophila* wing (*ptc-Gal4/*+). Ptc-Gal4 is expressed in the L3-L4 intervein region. At 22∼23°, when *dE2f1-dsRNA* (line #3) is expressed under control of *ptc-Gal4*, the L3-L4 intervein region is reduced by ∼50%, as shown in (B) (*w^1118^*; *ptc-Gal4*, *UAS-dE2f1dsRNA#3/*+; +/+). This wing phenotype can be strongly suppressed by *Df(2R)Exel6062* (C: *w^1118^*; *ptc-Gal4*, *UAS-dE2f1dsRNA#3/Df(2R)Exel6062*; +/+), or the *Su(z)2^1.a1^* allele (D: *w^1118^*; *ptc-Gal4*, *UAS-dE2f1dsRNA#3/Su(z)2^1.a1^*; +/+). The modification of the wing phenotype can be quantified by measuring the width of L3-L4 intervein region (E), and the genotypes of data presented in (E) are as follows: (*a*) *w^1118^*; *ptc-Gal4/*+; +; (*b*) *w^1118^*; *ptc-Gal4*, *UAS-dE2f1dsRNA#3/*+; +; (*c*) *w^1118^*; *ptc-Gal4*, *UAS-dE2f1dsRNA#3/Df(2R)Exel6062*; +/+; (*d*) *w^1118^*; *ptc-Gal4*, *UAS-dE2f1dsRNA#3/Su(z)2^1^*; +/+; (*e*) *w^1118^*; *ptc-Gal4*, *UAS-dE2f1dsRNA#3/Su(z)2^1.b7^*; +/+; (*f*) *w^1118^*; *ptc-Gal4*, *UAS-dE2f1dsRNA#3/Su(z)2^1.a1^*; +/+; and (*g*) *w^1118^*; *ptc-Gal4*, *UAS-dE2f1dsRNA#3/Su(z)2^k06344^*; +/+; (*h*) *w^1118^*; *ptc-Gal4*, *UAS-dE2f1dsRNA#3/*+; *Pc^f01890^/*+; (*i*) *w^1118^*; *ptc-Gal4*, *UAS-dE2f1dsRNA#3/*+; *Pc^3^/*+; (*j*) *w^1118^*; *ptc-Gal4*, *UAS-dE2f1dsRNA#3/*+; *His2AvD^810^/*+; and (*k*) *w^1118^*; *ptc-Gal4*, *UAS-dE2f1dsRNA#3/*+; *His2AvD^05146^/*+. At least 15 to 25 wings of each genotype (*a∼k*) were measured. Each genotype (*c∼k*) was compared with the control (*b*: *w^1118^*; *ptc-Gal4*, *UAS-dE2f1dsRNA#3/*+; +) and each comparison is highly significant (*P* < 4.9E-06 based on one-tailed *t*-test). For simplicity, “*ptc-Gal4*, *UAS-dE2f1dsRNA#3*” is referred as “PE3” in (E). The scale bar in (D) is 100µm.

One of the strongest suppressors (*Df(2R)Exel6062*) of the *dE2f1-dsRNA* phenotypes was reported to delete only one characterized gene, *Su(z)2* (Parks *et al.* 2004). *Df(2R)Exel6062* suppressed both the eye phenotype (Figure 2C compared to the control Figure 2B) and the wing phenotype (Figure 3C compared to the control Figure 3B). The *Df(2R)Exel6062* line deletes a region of ∼54kb between two *P*-element (XP vector) insertion lines *d09185* and *d02103* (Parks *et al.* 2004; Thibault *et al.* 2004). This deletion starts at 190bp region upstream of the neighboring gene *Posterior sex comb* (*Psc*), and includes *CG33798* (an uncharacterized gene with unknown function) and the *Su(z)2* gene (Parks *et al.* 2004).

To validate the suppressor gene of the *dE2f1-dsRNA* phenotypes, we tested the capacity of additional alleles of *Su(z)2* from the Bloomington stock center (Su*(z)2^1^*, *Su(z)2^1.a1^*, *Su(z)2^1.b7^*, *Su(z)2^1.b8^*, *Su(z)2^4d1^*, Su*(z)2^k06344^*) to modify the *dE2F1-RNAi* phenotypes (mutant alleles of *CG33798* are unavailable). These *Su(z)2* mutant alleles strongly suppressed the *dE2f1-dsRNA* (line #10) eye phenotype, and to a less extent with the strong eye phenotype generated by *dE2f1-dsRNA* (line #8; Figure 2E). Next, we validated these genetic interactions identified in the eye by testing the effect of *Su(z)2* mutants on the *dE2f1-dsRNA* wing phenotype. Reducing *Su(z)2* by either *Df(2R)Exel6062* (Figure 3C) or *Su(z)2^1.a1^* (Figure 3D) increased the L3-L4 intervein region of *ptc-Gal4 UAS-dE2f1-dsRNA* flies compared with controls (*ptc-Gal4 UAS-dE2f1-dsRNA/+*, Figure 3B). Measurement of L3-L4 distance demonstrated significant rescue of the intervein distance by these *Su(z)2* alleles compared to the control (Figure 3E). Together, these genetic analyses suggest that *Su(z)2* is a strong suppressor of *dE2f1-dsRNA* phenotypes.

We then sought to extend this observation by examining additional *Su(z)2* alleles described recently (Emmons *et al.* 2009). We examined the capacity of *Su(z)2* point mutant alleles (*Su(z)2^s15^*, *Su(z)2^s20^*, *Su(z)2^s21^*, *Su(z)2^s36^*, *Su(z)2^s84^*, *Su(z)2^s95^*, and *Su(z)2^sM^*) to suppress the *dE2f1-dsRNA* phenotypes. However, we did not observe any obvious modification of the *E2F1-dsRNA* phenotypes (data not shown), indicating that these particular *Su(z)2* point mutations are insufficient to modify these phenotypes. Similarly, we did not observe any genetic interactions between *dE2f1* and multiple alleles of the *Su(z)2* paralog, *Psc* (*Psc^s14^*, *Psc^e22^*, *Psc^h27^*, *Psc^EY06547^*, and *Psc^k07804^*; data not shown), suggesting that *Su(z)2* and *Psc* are not functionally redundant in these genetic analyses.

### Multiple PcG and PcG-related genes suppress the *dE2f1-dsRNA* phenotypes

Having identified *Su(z)2* as a suppressor of the *dE2f1-dsRNA* phenotypes, we tested whether mutants other than Polycomb family members, as well as genes that genetically interact with PcG, such as E(Pc) (Jürgens 1985; Campbell *et al.* 1995), Mi-2 (Kehle *et al.* 1998), and His2AvD (Swaminathan *et al.* 2005), could modify the *dE2f1-dsRNA* phenotypes. We observed that mutations of *Pc*, *pho*, *Su(z)12*, *Scm*, and *His2AvD* suppressed the *dE2f1-dsRNA* phenotypes, whereas *E(Pc)* and *Mi-2* behaved as enhancers (Table 3, Figure 3E). Importantly, components of three PcG complexes, including PRC 1 complex (*Pc*, *Scm*), PRC2 (*Esc*, *Su(z)12*), and PhoRC (*Pho*), were able to suppress the *dE2f1-dsRNA* phenotypes (Table 3), suggesting that PcG may repress dE2F1 activities. In addition, we observed that mutant alleles of several PcG/TrxG genes, such as *ash2^1^*, *crm^7^*, Dsp1^EP355^, *eff^8^*, *lid^10424^*, *lid^k06801^*, *Pcl^EY08457^*, *Sce^1^*, *trx^1^*, *trx^EY13717^*, showed variable genetic interactions ranging from suppression to no effect and enhancement of varied degrees (data not shown). These variable interactions might reflect the dynamic and complex interactions *in vivo*.

**Table 3:** Some of the PcG and TrxG genes dominantly modify the phenotypes caused by varied dE2F1 and RBF1 in the *Drosophila* eye and wing

Mutant Alleles	*GMR-Gal4,UAS-dE2f1-dsRNA#10**^a^*^,^*^b^*	*ptc-Gal4,UAS-dE2f1-dsRNA#3**^a^*^,^*^b^*	*Act88F-Gal4,UASdE2f**^a^*^,^*^b^*	*GMR-Gal4,UASdE2f1*, *UAS-dDp**^a^*^,^*^b^*
Suppressors				
* Asx^1^*	Suppression (5)	Suppression (1)	Enhancement (1)	NE
* eff^mer4^*	Suppression (5)	Suppression (2)	NE	NE
* E(Pc)84DE^T66.1^*	Suppression (5)	Suppression (1)	NE	NE
* esc^1^*	Suppression (4)	NE	ND	ND
* esc^21^*	Suppression (5)	Suppression (4)	ND	ND
* His2AvD^810^*	Suppression (5)	Suppression (2)	ND	Enhancement (3)
* His2AvD*^05146^	Suppression (5)	Suppression (3)	ND	Enhancement (3)
* Kis^BG01657^*	Suppression (5)	Suppression (2)	NE	NE
* Pc^3^*	Suppression (5)	Suppression (5)	ND	Enhancement (4)
* Pc^f01890^*	Suppression (5)	Suppression (2)	ND	Enhancement (1)
* pho^1^*	Suppression (5)	Suppression (1)	Enhancement (1)	NE
* Scm^D1^*	Suppression (5)	Suppression (4)	ND	ND
* Su(z)2^1^*	Suppression (5)	Suppression (4)	ND	Enhancement (1)
* Su(z)2^1.a1^*	Suppression (5)	Suppression (5)	NE	NE
* Su(z)2^1.b7^*	Suppression (5)	Suppression (3)	Enhancement (1)	NE
* Su(z)2^k06344^*	Suppression (5)	Suppression (3)	Enhancement (1)	NE
* Su(z)12^3^*	Suppression (5)	Suppression (2)	NE	NE
* tara^1^*	Suppression (5)	Suppression (1)	ND	NE
* tou*^2^	Suppression (5)	Suppression (2)	Enhancement	NE
* brm^2^*	Suppression (5)	Suppression (2)	NE	NE
* trx^KG08639^*	Suppression (5)	Suppression (1)	NE	NE
Enhancers				
* E(Pc)^w3^*	Enhancement (4)	Enhancement (3)	Suppression (1)	ND
* E(Pc)^D4^*	Enhancement (5)	Enhancement (5)	Suppression (1)	ND
* Mi-2^j3D4^*	Enhancement (4)	Enhancement (1)	Suppression (4)	NE
* Mi-2^EY08138^*	Enhancement (5)	Enhancement (1)	ND	ND
* Su(z)3^1^*	Enhancement (5)	Enhancement (4)	Suppression (1)	ND
* tara^BG01673^*	Pupal lethal	Enhancement (4)	Lethal	Lethal

a The effects of suppression or enhancement were ranked with scores from 1 to 5, with “1” the weakest and “5” the strongest; NE, no effect; ND, not determined.

b These crosses were maintained at 25°.

c These crosses were maintained at 22-23°; see *Materials and Methods* for the detailed genotypes analyzed.

Next, we tested whether PcG mutants could modify phenotypes caused by overexpression of *dE2f1* alone or together with *dDp*, as we described previously (Staehling-Hampton *et al.* 1999; Morris *et al.* 2008). We found that PcG mutants weakly enhanced phenotypes associated with *dE2f1* overexpression (Table 3), which is consistent with the PcG role in repressing dE2F1 activities. Furthermore, to examine whether the PcG genes affect RNAi efficiency, we used the *GMR-w^IR^* line and tested several PcG mutants, including *E(Pc)^w3^*, *Psc^1^*, *Psc^e23^*, *Psc^e25^*, *Psc^h28^*, *Su(z)2^1.b8^*, *Su(z)2^k06344^*, *Su(z)2^4d1^*, *Su(z)2^s15^*, *Su(z)2^s20^*, *Su(z)2^s95^*, and *Su(z)2^sM^*. We did not observe any of these lines affected the light yellow eye color caused by knocking down of *white* gene (data not shown), suggesting that *Psc* and *Su(z)2* does not affect RNAi process. Taken together, these genetic analyses revealed *in vivo* regulation of dE2F1 by the PcG complexes, suggesting that several PcG complexes cooperate to restrict dE2F1-dependent cell proliferation.

### Su(z)2 represses the expression of *dE2f1* and critical proliferation target genes

**Figure 4:**
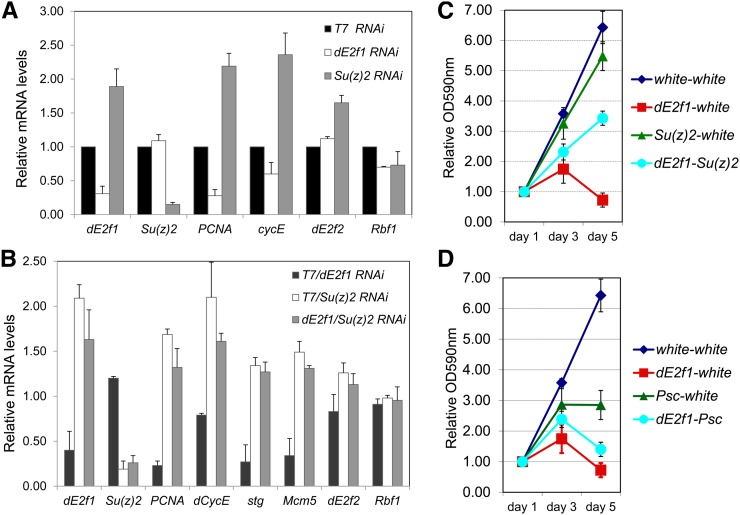
Su(z)2 regulates the transcription of *dE2f1* and some of the dE2F1 target genes. (A) Knocking down *Su(z)2* (gray bars) leads to up-regulation of *dE2f1*, and some of the dE2F1 target genes, such as *PCNA*, *dCycE*, and to a less extent *dE2f2* and no effect of *Rbf1*, based on qRT-PCR assay. The samples treated with *dE2f1-dsRNAs* (white bars) serve as a positive control, and *T7-dsRNA* treated samples are negative controls. (B) Codepletion (gray bars) of *Su(z)2* and *dE2f1* suppresses the effect of *dE2f1-dsRNA* treatment and leads to increased expression of *dE2f1*, *PCNA*, *dCycE*, *stg*, and *mcm5*. The total dsRNAs are normalized with *T7*-dsRNA. (C and D) Effect of dsRNA treatment of the growth of SL2 cells: knocking down of *Su(z)2* (C), but not Psc (D), suppresses the effect of *dE2f1-dsRNA* treatment at day 5. For each sample, the total amount of dsRNA is normalized with *white*-dsRNA and cell viability was determined by using the dimethyltriazoldiphenyl tetrazolium-formazan assay after 1, 3 or 5 days of dsRNA treatment.

To examine the role of Su(z)2 in regulating dE2F1 activity, we depleted *Su(z)2* in cultured *Drosophila* SL2 cells and analyzed the expression of *dE2f1* and a subset of critical proliferation target genes by qRT-PCR. Depletion of *Su(z)2* significantly increased the transcription of *dE2f1* and dE2F1 target genes including *PCNA* and *dCycE* (Figure 4A). In contrast, reduction of *Su(z)2* had little effect on *Rbf1* transcription and weakly up-regulates the expression of *dE2f2* gene (Figure 4A). These results suggest that Su(z)2 constrains cell proliferation by regulating the expression of *dE2f1*, *PCNA*, and *dCycE*.

To test whether depletion of *Su(z)2* could rescue the effect of reduced *dE2f1* transcription, we codepleted *Su(z)2* and *dE2f1* in *Drosophila* SL2 cells and measured the effect on dE2F target gene expression. As shown in Figure 4B, we observed that compared to knocking down *dE2f1* alone, codepletion of Su(z)2 and dE2F1 significantly increased the expression of *dE2f1* and several dE2F1 target genes, including *PCNA*, *dCycE*, *string* (*stg*, encoding *Drosophila* CDC25 phosphatase), and *Mcm5*. These results suggest that reduction of *Su(z)2* is sufficient to alleviate the effect of *dE2f1* depletion in SL2 cells, which is consistent with our observations that *Su(z)2* mutants can suppress the *dE2f1-dsRNA* phenotypes.

Next, to determine the biological consequence of codepleting *dE2f1* and *Su(z)2*, we conducted the dimethyltriazoldiphenyl tetrazolium-formazan cell viability assays, also known as the MTT assay, to analyze the kinetics of cell proliferation in SL2 cells. This assay is based on mitochondrial reduction of a tetrazolium salt to a colored formazan salt, which can be quantified by measuring the absorbance at 570 nm, in living cells (Hansen *et al.* 1989). Depletion of *dE2f1* impairs cellular proliferation, and cells arrest after 5 days of dsRNA treatment (Figure 4C). Reducing *Su(z)2* levels alone has little effect on cell proliferation (Figure 4C); however, codepletion of *dE2f1* and *Su(z)2* significantly rescues the proliferation defects associated with *dE2f1* depletion. In contrast, codepletion of *dE2f1* and *Psc* (or *E(Pc)*; data not shown) had no effect in rescue of this defect (Figure 4D), which is consistent with our genetic analyses (Table 3). Interestingly, depleting Psc alone blocked cell proliferation (Figure 4D), consistent with the recently reported role of Psc in regulating the G2-M progression by directly affecting Cyclin B degradation (Mohd-Sarip *et al.* 2012). In contrast to Psc, depleting Su(z)2 does not affect cell proliferation (Figure 4C), suggesting that unlike Psc, Su(z)2 may not regulate the turnover of CycB and nuclear division. Taken together, these results suggest that Su(z)2 represses the transcription of *dE2f1* and certain dE2F1 target genes that are required for cell proliferation.

## Discussion

PcG and TrxG complexes play important roles in maintaining the expression of many developmental genes in metazoans, and deregulation of their functions has been linked to human malignancy. Here we identify genetic interactions between multiple components of PcG complexes and a key cell-cycle regulator, E2F1, in *Drosophila*. We find that mutations compromising the PcG functions suppress the defects caused by dE2F1-RNAi in the *Drosophila* eye and wing. Our results suggest that PcG complexes may regulate the key cell-cycle regulator dE2F1 and a subset of dE2F1 target genes in *Drosophila* development. To our knowledge, this is the first work to show functionally that dE2F1 is affected by PcG proteins, especially by Su(z)2.

### Mutant alleles of *Su(z)2*, but not *Psc*, suppress *dE2f1-dsRNA* phenotypes

Our dominant modifier genetic screen using Exelixis *Df* mutants identified *Su(z)2* as a strong suppressor of the *dE2F1-RNAi* phenotypes. By expanding our studies to mutations of other components of the PcG complexes, we found a strong genetic link between PcG and E2F1 activity. However, as summarized in Table 3, not all of mutant alleles of the PcG genes tested modified the *dE2f1-dsRNA* phenotypes. For example, although Su(z)2 and Psc are paralogs and their functions are partially redundant (Brunk *et al.* 1991; van Lohuizen *et al.* 1991; Soto *et al.* 1995; Wu and Howe 1995; Stankunas *et al.* 1998), we found that only Su(z)2 could modify the E2F1-RNAi phenotypes. In addition, biochemical analyses suggest that both Psc and Su(z)2 share similar activities in DNA binding, chromatin compacting, and chromatin remodeling inhibition (Lo *et al.* 2009). However, in multiple analyses, including genetic tests based on phenotypes caused by overexpression or knockdown of dE2F1, and experiments in cultured SL2 cells, we observed a consistent pattern of interaction with *Su(z)2* but not *Psc* (Figure 2, Figure 4, Table 3).

There are several potential explanations to these observations. First, this screen was designed to identify the dominant modifiers and perhaps mutations within some PcG genes remain above a critical threshold during development. Second, the *dE2f1-dsRNA* phenotypes in both the eye and wing are caused by reduction of dE2F1 protein levels and dE2F1 activity (Morris *et al.* 2008). Because dE2F1 levels vary during the cell cycle (Shibutani *et al.* 2008), the dynamic interactions between dE2F1 and PcG gene products may determine whether a phenotypic interaction can be visualized in these adult tissues. Perhaps Su(z)2 has a more important role in the tissues we used to screen for E2F1 modifiers, and our genetic tests alone still cannot rigorously rule out the potential redundant functions of Su(z)2 and Psc. Third, as Psc regulates mitotic progression independently of the transcriptional functions of the canonical PcG complexes, it is likely that Su(z)2 and Psc regulate different sets of targets (Mohd-Sarip *et al.* 2012). Unlike Psc (Figure 4D), depleting Su(z)2 alone does not affect cell proliferation (Figure 4C), suggesting that Su(z)2 may not have a role in regulating CycB degradation. Nevertheless, the mitotic effects of Psc may mask its role in regulating *dE2f1* transcription. Thus, our results are not sufficient to exclude the possibility that Psc might have a redundant role with Su(z)2 in repressing the expression of *dE2f1*. Additional molecular and biochemical analyses are necessary to further dissect the difference between these two paralog proteins.

### The *dE2f1* gene is a target repressed by PcG complexes

There are several lines of evidence suggesting that dE2F1 activity is regulated by PcG and TrxG complexes. Mutant alleles of subunits of the SWI/SNF chromatin-remodeling complex (Grimaud *et al.* 2006), such as *brahma* (*brm*) and *moira* (*mor*), have been shown to dominantly modify the rough eye phenotype caused by overexpression of *dE2f1* and its heterodimeric partner *dDp* (Staehling-Hampton *et al.* 1999). Subunits of the Domino chromatin-remodeling complex (PcG-like L3mbt and the related dSfmbt) negatively regulate transcription of an artificial *dE2f1* reporter gene (Lu *et al.* 2007). ChIP assays have identified both Ph and Pho on the promoter and coding regions of the *dE2f1* gene in *Drosophila* embryos (Oktaba *et al.* 2008).

PcG complexes regulate methylation of H3K27 in *Drosophila* (Cao and Zhang 2004), we therefore analyzed the status of H3K27 methylation during development or in several *Drosophila* cell lines using chromatin immunoprecipitation (ChIP) followed by microarray hybridization (ChIP-chip) or high-throughput sequencing (ChIP-Seq) data sets deposited to modENCODE (Celniker *et al.* 2009) (http://modencode.oicr.on.ca/fgb2/gbrowse/fly/). We found that the genomic loci of *dE2f1*, *dCycE* and *stg* display mono-, di-, or trimethylation of H3K27 (H3K27me1/2/3) during development or in *Drosophila* cell lines, including SL2, Kc, and BG3 cells (see Figure S1, Figure S2, Figure S3, and Figure S4 for details), suggesting that PcG may directly regulate the expression of these genes. *dCycE* and *stg* are critical dE2F1 target genes, which regulate the G1/S-phase and the G2/M-phase transition of the cell cycle, respectively (Edgar and Lehner 1996; Dyson 1998). We did not observe obvious H3K27me modification of other dE2F1 target genes such as *PCNA* and *Mcm5* (data not shown), suggesting that the effect of Su(z)2 on expression of these genes (Figure 4B) is likely indirect through *dE2f1*. Together, these observations suggest that PcG complexes may repress the expression of *dE2f1* and a subset of dE2F1 target genes during development.

These observations are consistent with our genetic studies and suggest the suppressive effect of PcG mutants on *dE2f1-dsRNA* phenotypes is caused by derepression of *dE2f1* and certain dE2F1 target genes, which compensates for the effect of *dE2f1*-depletion. Together with previous published observations linking PcG complexes to cell-cycle regulators, such as *dCycA* (Martinez *et al.* 2006), *dCycB* (Oktaba *et al.* 2008), *dCycE* (Brumby *et al.* 2002), and *dE2f1* (Oktaba *et al.* 2008), our observations provide further support for the role of PcG in repressing the transcription of cell-cycle genes, including *dE2f1*, *dCycE*, and *stg* (Figure 4 and Figure S4).

Regulation of the key cell-cycle regulators by PcG complexes may present a general mechanism to coordinate cellular differentiation and proliferation during development. Disrupting the coordination between differentiation and proliferation may result in abnormal development and may contribute to tumorigenesis. Consistent with this notion, accumulating evidence shows that the PcG complexes are misregulated in a wide variety of human cancers (Sparmann and van Lohuizen 2006; Ballestar and Esteller 2008; Bracken and Helin 2009). This study, together with previous reports in *Drosophila* (Staehling-Hampton *et al.* 1999; Brumby *et al.* 2002; Grimaud *et al.* 2006; Martinez *et al.* 2006; Lu *et al.* 2007; Oktaba *et al.* 2008), suggest that mutations compromising PcG activity would elevate E2F activity, thereby providing cells with a strong tumorigenic advantage. Further studies are necessary to elucidate how these two important regulatory mechanisms are coordinated during cellular differentiation and proliferation in development.

## Supplementary Material

Supporting Information

## References

[bib1] Attwooll C. Oddi S. Cartwright P. Prosperini E. Agger K., 2005 A novel repressive E2F6 complex containing the polycomb group protein, EPC1, that interacts with EZH2 in a proliferation-specific manner. J. Biol. Chem. 280: 1199–1208 15536069 10.1074/jbc.M412509200

[bib2] Ballestar E. Esteller M., 2008 Epigenetic gene regulation in cancer. Adv. Genet. 61: 247–267 18282509 10.1016/S0065-2660(07)00009-0

[bib3] Bracken A. P. Helin K., 2009 Polycomb group proteins: navigators of lineage pathways led astray in cancer. Nat. Rev. Cancer 9: 773–784 19851313 10.1038/nrc2736

[bib4] Bracken A. P. Pasini D. Capra M. Prosperini E. Colli E., 2003 EZH2 is downstream of the pRB-E2F pathway, essential for proliferation and amplified in cancer. EMBO J. 22: 5323–5335 14532106 10.1093/emboj/cdg542 PMC213796

[bib5] Brand A. H. Manoukian A. S. Perrimon N., 1994 Ectopic expression in Drosophila. Methods Cell Biol. 44: 635–654 7707973 10.1016/s0091-679x(08)60936-x

[bib6] Brumby A. M. Zraly C. B. Horsfield J. A. Secombe J. Saint R., 2002 Drosophila cyclin E interacts with components of the Brahma complex. EMBO J. 21: 3377–3389 12093739 10.1093/emboj/cdf334 PMC126084

[bib7] Brunk B. P. Martin E. C. Adler P. N., 1991 Drosophila genes Posterior Sex Combs and Suppressor two of zeste encode proteins with homology to the murine bmi-1 oncogene. Nature 353: 351–353 1833647 10.1038/353351a0

[bib8] Burkhart D. L. Sage J., 2008 Cellular mechanisms of tumour suppression by the retinoblastoma gene. Nat. Rev. Cancer 8: 671–682 18650841 10.1038/nrc2399 PMC6996492

[bib9] Campbell R. B. Sinclair D. A. Couling M. Brock H. W., 1995 Genetic interactions and dosage effects of Polycomb group genes of Drosophila. Mol. Gen. Genet. 246: 291–300 7854314 10.1007/BF00288601

[bib10] Cao R. Zhang Y., 2004 The functions of E(Z)/EZH2-mediated methylation of lysine 27 in histone H3. Curr. Opin. Genet. Dev. 14: 155–164 15196462 10.1016/j.gde.2004.02.001

[bib11] Cao R. Wang L. Wang H. Xia L. Erdjument-Bromage H., 2002 Role of histone H3 lysine 27 methylation in Polycomb-group silencing. Science 298: 1039–1043 12351676 10.1126/science.1076997

[bib12] Castelli-Gair J. E. Micol J. L. Garcia-Bellido A., 1990 Transvection in the Drosophila Ultrabithorax gene: a Cbx1 mutant allele induces ectopic expression of a normal allele in trans. Genetics 126: 177–184 2121595 10.1093/genetics/126.1.177 PMC1204122

[bib13] Celniker S. E. Dillon L. A. Gerstein M. B. Gunsalus K. C. Henikoff S., 2009 Unlocking the secrets of the genome. Nature 459: 927–930 19536255 10.1038/459927a PMC2843545

[bib14] Courel M. Friesenhahn L. Lees J. A., 2008 E2f6 and Bmi1 cooperate in axial skeletal development. Dev. Dyn. 237: 1232–1242 18366140 10.1002/dvdy.21516 PMC2697036

[bib15] Dahiya A. Wong S. Gonzalo S. Gavin M. Dean D. C., 2001 Linking the Rb and polycomb pathways. Mol. Cell 8: 557–569 11583618 10.1016/s1097-2765(01)00346-x

[bib16] Dimova D. K. Stevaux O. Frolov M. V. Dyson N. J., 2003 Cell cycle-dependent and cell cycle-independent control of transcription by the Drosophila E2F/RB pathway. Genes Dev. 17: 2308–2320 12975318 10.1101/gad.1116703 PMC196467

[bib17] Duronio R. J. O’Farrell P. H. Xie J. E. Brook A. Dyson N., 1995 The transcription factor E2F is required for S phase during Drosophila embryogenesis. Genes Dev. 9: 1445–1455 7601349 10.1101/gad.9.12.1445

[bib18] Dyson N., 1998 The regulation of E2F by pRB-family proteins. Genes Dev. 12: 2245–2262 9694791 10.1101/gad.12.15.2245

[bib19] Edgar B. A. Lehner C. F., 1996 Developmental control of cell cycle regulators: a fly’s perspective. Science 274: 1646–1652 8939845 10.1126/science.274.5293.1646

[bib20] Emmons R. B. Genetti H. Filandrinos S. Lokere J. Wu C. T., 2009 Molecular genetic analysis of Suppressor 2 of zeste identifies key functional domains. Genetics 182: 999–1013 19528329 10.1534/genetics.108.097360 PMC2728886

[bib21] Fischle W. Wang Y. Jacobs S. A. Kim Y. Allis C. D., 2003 Molecular basis for the discrimination of repressive methyl-lysine marks in histone H3 by Polycomb and HP1 chromodomains. Genes Dev. 17: 1870–1881 12897054 10.1101/gad.1110503 PMC196235

[bib22] Frolov M. V. Dyson N. J., 2004 Molecular mechanisms of E2F-dependent activation and pRB-mediated repression. J. Cell Sci. 117: 2173–2181 15126619 10.1242/jcs.01227

[bib23] Gil J. Peters G., 2006 Regulation of the INK4b-ARF-INK4a tumour suppressor locus: all for one or one for all. Nat. Rev. Mol. Cell Biol. 7: 667–677 16921403 10.1038/nrm1987

[bib24] Grimaud C. Negre N. Cavalli G., 2006 From genetics to epigenetics: the tale of Polycomb group and trithorax group genes. Chromosome Res. 14: 363–375 16821133 10.1007/s10577-006-1069-y

[bib25] Hannon G. J., 2002 RNA interference. Nature 418: 244–251 12110901 10.1038/418244a

[bib26] Hansen M. B. Nielsen S. E. Berg K., 1989 Re-examination and further development of a precise and rapid dye method for measuring cell growth/cell kill. J. Immunol. Methods 119: 203–210 2470825 10.1016/0022-1759(89)90397-9

[bib27] Jürgens G., 1985 A group of genes controlling the spatial expression of the bithorax complex in Drosophila. Nature 316: 153–155

[bib28] Kehle J. Beuchle D. Treuheit S. Christen B. Kennison J. A., 1998 dMi-2, a hunchback-interacting protein that functions in polycomb repression. Science 282: 1897–1900 9836641 10.1126/science.282.5395.1897

[bib29] Lee Y. S. Carthew R. W., 2003 Making a better RNAi vector for Drosophila: use of intron spacers. Methods 30: 322–329 12828946 10.1016/s1046-2023(03)00051-3

[bib30] Lee Y. S. Nakahara K. Pham J. W. Kim K. He Z., 2004 Distinct roles for Drosophila Dicer-1 and Dicer-2 in the siRNA/miRNA silencing pathways. Cell 117: 69–81 15066283 10.1016/s0092-8674(04)00261-2

[bib31] Levine S. S. King I. F. Kingston R. E., 2004 Division of labor in polycomb group repression. Trends Biochem. Sci. 29: 478–485 15337121 10.1016/j.tibs.2004.07.007

[bib32] Lo S. M. Ahuja N. K. Francis N. J., 2009 Polycomb group protein Suppressor 2 of zeste is a functional homolog of Posterior Sex Combs. Mol. Cell. Biol. 29: 515–525 18981224 10.1128/MCB.01044-08 PMC2612506

[bib33] Lu J. Ruhf M. L. Perrimon N. Leder P., 2007 A genome-wide RNA interference screen identifies putative chromatin regulators essential for E2F repression. Proc. Natl. Acad. Sci. USA 104: 9381–9386 17517653 10.1073/pnas.0610279104 PMC1890503

[bib34] Margueron R. Reinberg D., 2011 The Polycomb complex PRC2 and its mark in life. Nature 469: 343–349 21248841 10.1038/nature09784 PMC3760771

[bib35] Martinez A. M. Colomb S. Dejardin J. Bantignies F. Cavalli G., 2006 Polycomb group-dependent Cyclin A repression in Drosophila. Genes Dev. 20: 501–513 16481477 10.1101/gad.357106 PMC1369051

[bib36] Min J. Zhang Y. Xu R. M., 2003 Structural basis for specific binding of Polycomb chromodomain to histone H3 methylated at Lys 27. Genes Dev. 17: 1823–1828 12897052 10.1101/gad.269603 PMC196225

[bib37] Mohd-Sarip A. Lagarou A. Doyen C. M. van der Knaap J. A. Aslan U., 2012 Transcription-independent function of Polycomb group protein PSC in cell cycle control. Science 336: 744–747 22491092 10.1126/science.1215927

[bib38] Morris E. J. Ji J. Y. Yang F. Di Stefano L. Herr A., 2008 E2F1 represses beta-catenin transcription and is antagonized by both pRB and CDK8. Nature 455: 552–556 18794899 10.1038/nature07310 PMC3148807

[bib39] Müller H. Helin K., 2000 The E2F transcription factors: key regulators of cell proliferation. Biochim. Biophys. Acta 1470: M1–M12 10656985 10.1016/s0304-419x(99)00030-x

[bib40] Müller J. Verrijzer P., 2009 Biochemical mechanisms of gene regulation by polycomb group protein complexes. Curr. Opin. Genet. Dev. 19: 150–158 19345089 10.1016/j.gde.2009.03.001

[bib41] Ogawa H. Ishiguro K. Gaubatz S. Livingston D. M. Nakatani Y., 2002 A complex with chromatin modifiers that occupies E2F- and Myc-responsive genes in G0 cells. Science 296: 1132–1136 12004135 10.1126/science.1069861

[bib42] Oktaba K. Gutierrez L. Gagneur J. Girardot C. Sengupta A. K., 2008 Dynamic regulation by polycomb group protein complexes controls pattern formation and the cell cycle in Drosophila. Dev. Cell 15: 877–889 18993116 10.1016/j.devcel.2008.10.005

[bib43] Parks A. L. Cook K. R. Belvin M. Dompe N. A. Fawcett R., 2004 Systematic generation of high-resolution deletion coverage of the Drosophila melanogaster genome. Nat. Genet. 36: 288–292 14981519 10.1038/ng1312

[bib44] Ren B. Cam H. Takahashi Y. Volkert T. Terragni J., 2002 E2F integrates cell cycle progression with DNA repair, replication, and G(2)/M checkpoints. Genes Dev. 16: 245–256 11799067 10.1101/gad.949802 PMC155321

[bib45] Schuettengruber B. Chourrout D. Vervoort M. Leblanc B. Cavalli G., 2007 Genome regulation by polycomb and trithorax proteins. Cell 128: 735–745 17320510 10.1016/j.cell.2007.02.009

[bib46] Schwartz Y. B. Pirrotta V., 2007 Polycomb silencing mechanisms and the management of genomic programmes. Nat. Rev. Genet. 8: 9–22 17173055 10.1038/nrg1981

[bib47] Sherr C. J., 2004 Principles of tumor suppression. Cell 116: 235–246 14744434 10.1016/s0092-8674(03)01075-4

[bib48] Shibutani S. T. de la Cruz A. F. Tran V. Turbyfill W. J. 3rd Reis T., 2008 Intrinsic negative cell cycle regulation provided by PIP box- and Cul4Cdt2-mediated destruction of E2f1 during S phase. Dev. Cell 15: 890–900 19081076 10.1016/j.devcel.2008.10.003 PMC2644461

[bib49] Soto M. C. Chou T. B. Bender W., 1995 Comparison of germline mosaics of genes in the Polycomb group of Drosophila melanogaster. Genetics 140: 231–243 7635288 10.1093/genetics/140.1.231 PMC1206550

[bib50] Sparmann A. van Lohuizen M., 2006 Polycomb silencers control cell fate, development and cancer. Nat. Rev. Cancer 6: 846–856 17060944 10.1038/nrc1991

[bib51] Staehling-Hampton K. Ciampa P. J. Brook A. Dyson N., 1999 A genetic screen for modifiers of E2F in Drosophila melanogaster. Genetics 153: 275–287 10471712 10.1093/genetics/153.1.275 PMC1460754

[bib52] Stankunas K. Berger J. Ruse C. Sinclair D. A. Randazzo F., 1998 The enhancer of polycomb gene of Drosophila encodes a chromatin protein conserved in yeast and mammals. Development 125: 4055–4066 9735366 10.1242/dev.125.20.4055

[bib53] Stevaux O. Dyson N. J., 2002 A revised picture of the E2F transcriptional network and RB function. Curr. Opin. Cell Biol. 14: 684–691 12473340 10.1016/s0955-0674(02)00388-5

[bib54] Storre J. Elsasser H. P. Fuchs M. Ullmann D. Livingston D. M., 2002 Homeotic transformations of the axial skeleton that accompany a targeted deletion of E2f6. EMBO Rep. 3: 695–700 12101104 10.1093/embo-reports/kvf141 PMC1084195

[bib55] Swaminathan J. Baxter E. M. Corces V. G., 2005 The role of histone H2Av variant replacement and histone H4 acetylation in the establishment of Drosophila heterochromatin. Genes Dev. 19: 65–76 15630020 10.1101/gad.1259105 PMC540226

[bib56] Thacker S. A. Bonnette P. C. Duronio R. J., 2003 The contribution of E2F-regulated transcription to Drosophila PCNA gene function. Curr. Biol. 13: 53–58 12526745 10.1016/s0960-9822(02)01400-8

[bib57] Thibault S. T. Singer M. A. Miyazaki W. Y. Milash B. Dompe N. A., 2004 A complementary transposon tool kit for Drosophila melanogaster using P and piggyBac. Nat. Genet. 36: 283–287 14981521 10.1038/ng1314

[bib58] Trimarchi J. M. Lees J. A., 2002 Sibling rivalry in the E2F family. Nat. Rev. Mol. Cell Biol. 3: 11–20 11823794 10.1038/nrm714

[bib59] Trimarchi J. M. Fairchild B. Wen J. Lees J. A., 2001 The E2F6 transcription factor is a component of the mammalian Bmi1-containing polycomb complex. Proc. Natl. Acad. Sci. USA 98: 1519–1524 11171983 10.1073/pnas.041597698 PMC29289

[bib60] van den Heuvel S. Dyson N. J., 2008 Conserved functions of the pRB and E2F families. Nat. Rev. Mol. Cell Biol. 9: 713–724 18719710 10.1038/nrm2469

[bib61] van Lohuizen M. Frasch M. Wientjens E. Berns A., 1991 Sequence similarity between the mammalian bmi-1 proto-oncogene and the Drosophila regulatory genes Psc and Su(z)2. Nature 353: 353–355 1922340 10.1038/353353a0

[bib62] Wu C. T. Howe M., 1995 A genetic analysis of the Suppressor 2 of zeste complex of Drosophila melanogaster. Genetics 140: 139–181 7635282 10.1093/genetics/140.1.139 PMC1206544

[bib63] Zhao X. Feng D. Wang Q. Abdulla A. Xie X. J., 2012 Regulation of lipogenesis by cyclin-dependent kinase 8-mediated control of SREBP-1. J. Clin. Invest. 122: 2417–2427 22684109 10.1172/JCI61462 PMC3386818

