# Supplementary material for: In Vivo Regulation of E2F1 by Polycomb Group Genes in Drosophila
Source: G3 (Bethesda). 2012 Dec 1;2(12):1651–60. doi: 10.1534/g3.112.004333 (PMC3516486; doi:10.1534/g3.112.004333)
Supplement: Supporting Information [file supp_2_12_1651__index.html]

Supporting Information 

# *In Vivo* Regulation of E2F1 by Polycomb Group Genes in *Drosophila*

## Supporting Information for Ji *et al.*, 2012

**Files in this Data Supplement:**

- Supporting Information - Figures S1-S4 and Table S1 (PDF, 2.6 MB)
- Figure S1 - The H3K27me status on the *dE2f1* gene (PDF, 748 KB)
- Figure S2 - The H3K27me status on the *dCycE* gene (PDF, 805 KB)
- Figure S3 - The H3K27me status on the *stg* gene (PDF, 765 KB)
- Figure S4 - The detailed results of ChIP-Seq analyses showing the enrichment of H3K27me3 in *dE2f1* (PDF, 423 KB)
- Table S1 - Summary of the genetic analyses using the Exelixis *Df* lines (.xls, 255 KB)
