# Supplementary material for: In Vivo Regulation of E2F1 by Polycomb Group Genes in Drosophila
Source: G3 (Bethesda). 2012 Dec 1;2(12):1651–60. doi: 10.1534/g3.112.004333 (PMC3516486; doi:10.1534/g3.112.004333)
Supplement: Supporting Information [file supp_2.12.1651_004333SI.pdf]

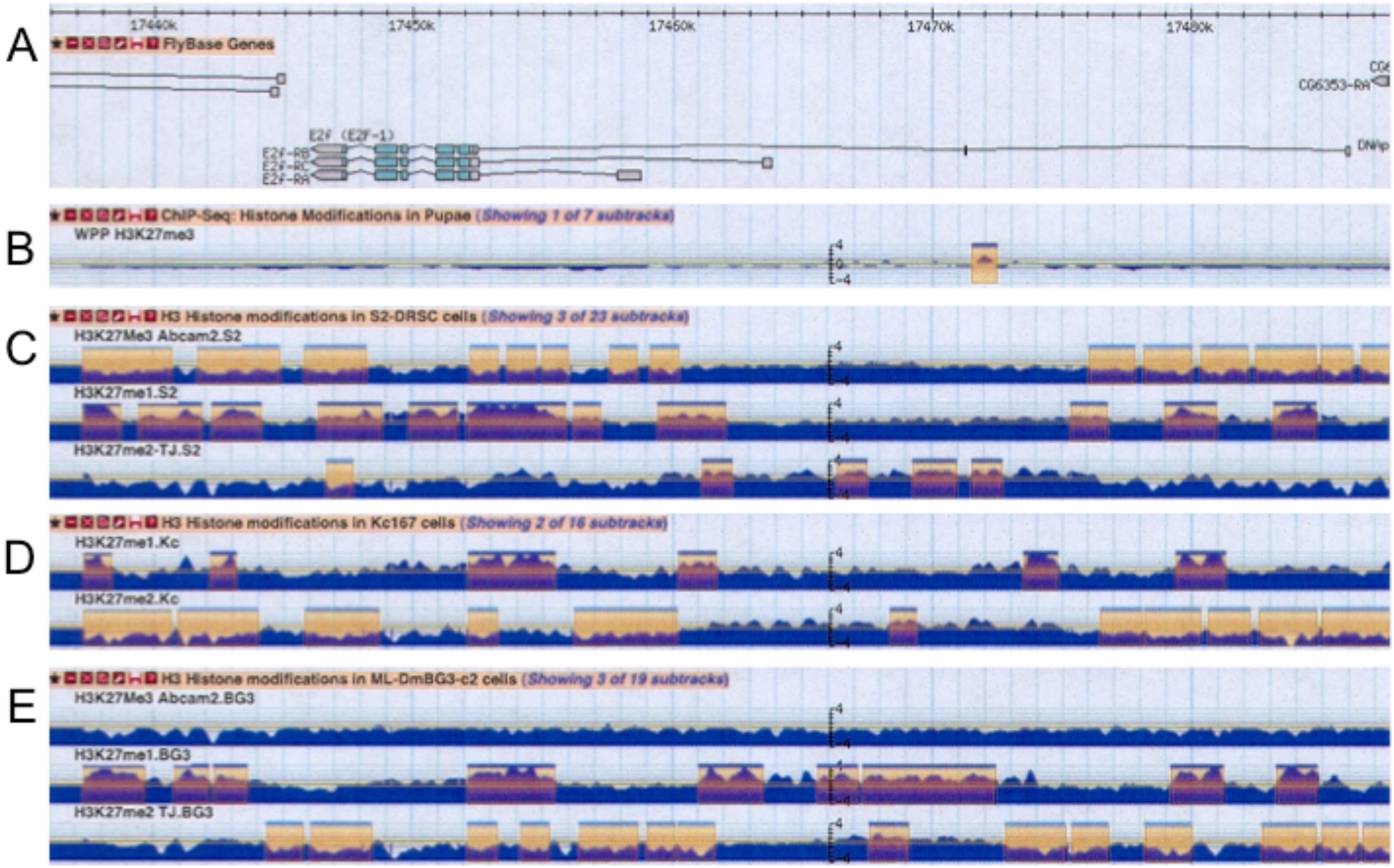

Figure S1 The H3K27me status on the *dE2f1*gene (**A**; Region surveyed: 3R:17444026..17488127) during the white prepupal (WPP) stage (**B**) and in *Drosophila* SL2 (**C**), Kc (**D**) and BG3 (**E**) cells. These results are obtained from the ChIP-chip and ChIP-Seq data sets provided by Drs. Gary Karpen, Vincenzo Pirrotta, and Kevin White laboratories from the modENCODE Project (http://modencode.oicr.on.ca/fgb2/gbrowse/fly/). Note: H3K27me3 is enriched at the *dE2f1* locus during the WPP developmental stage (**B**), and the enrichment of H3K27me1/2 in *dE2f1* locus in all the three cell lines analyzed (**C/D/E**).

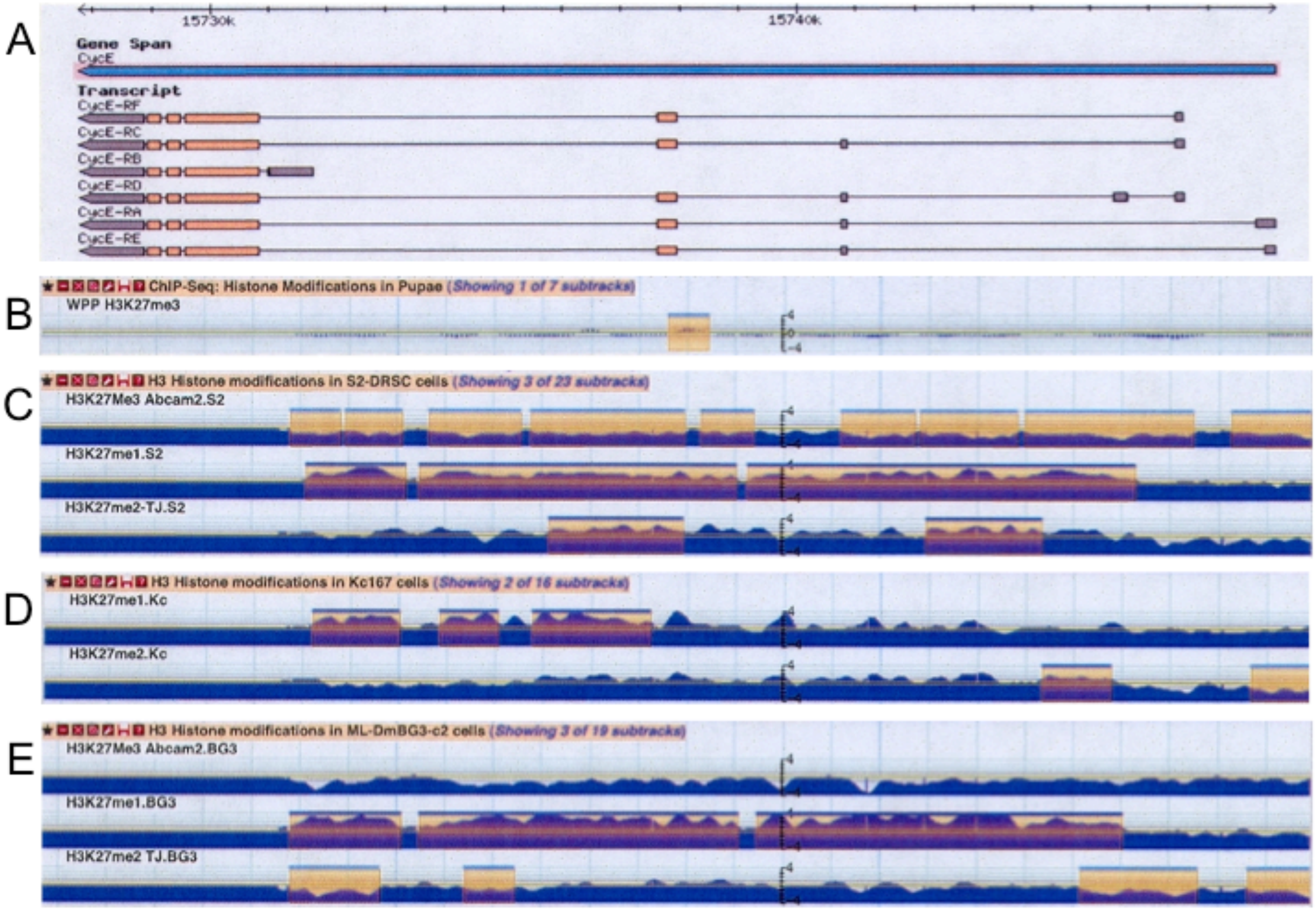

Figure S2 The H3K27me status on the *dCycE* gene (**A**; Region surveyed: 2L:15712959..15762958) during the WPP stage (**B**) and in *Drosophila* SL2 (**C**), Kc (**D**) and BG3 (**E**) cells. Note the enrichment of H3K27me3 in *dCycE* locus during the WPP stage (**B**), and the enrichment of H3K27me1/2 in *dCycE* locus in all the three cell lines analyzed (**C/D/E**).

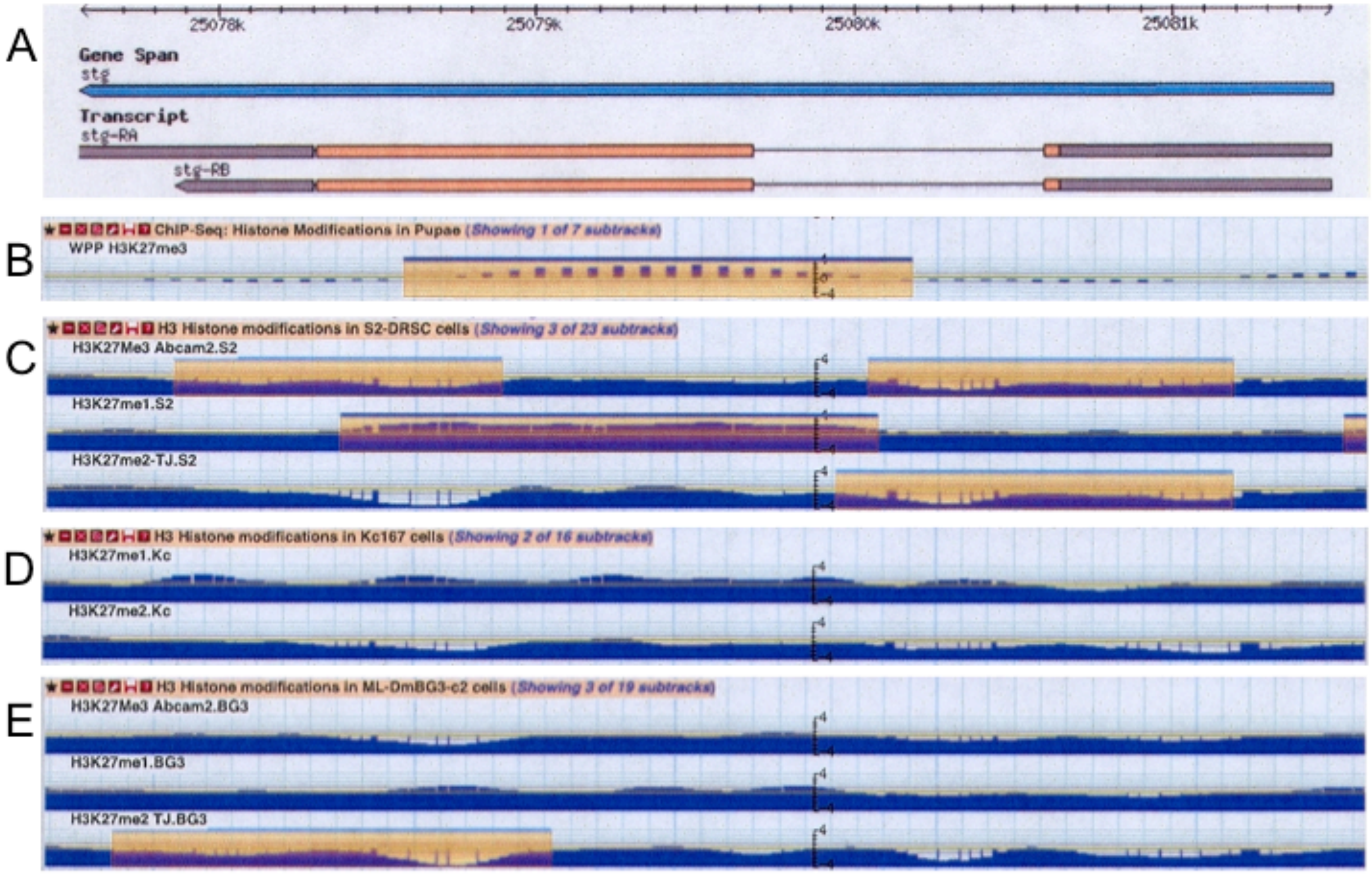

Figure S3 The H3K27me status on the *stg* gene (**A**; Region surveyed: 3R:25064521..25114520) during the WPP stage (**B**) and in *Drosophila* SL2 (**C**), Kc (**D**) and BG3 (**E**) cells. Note the enrichment of H3K27me3 in *stg* locus during the WPP stage (**B**), and the enrichment of H3K27me1 in *stg* locus in SL2 cells (**C**).

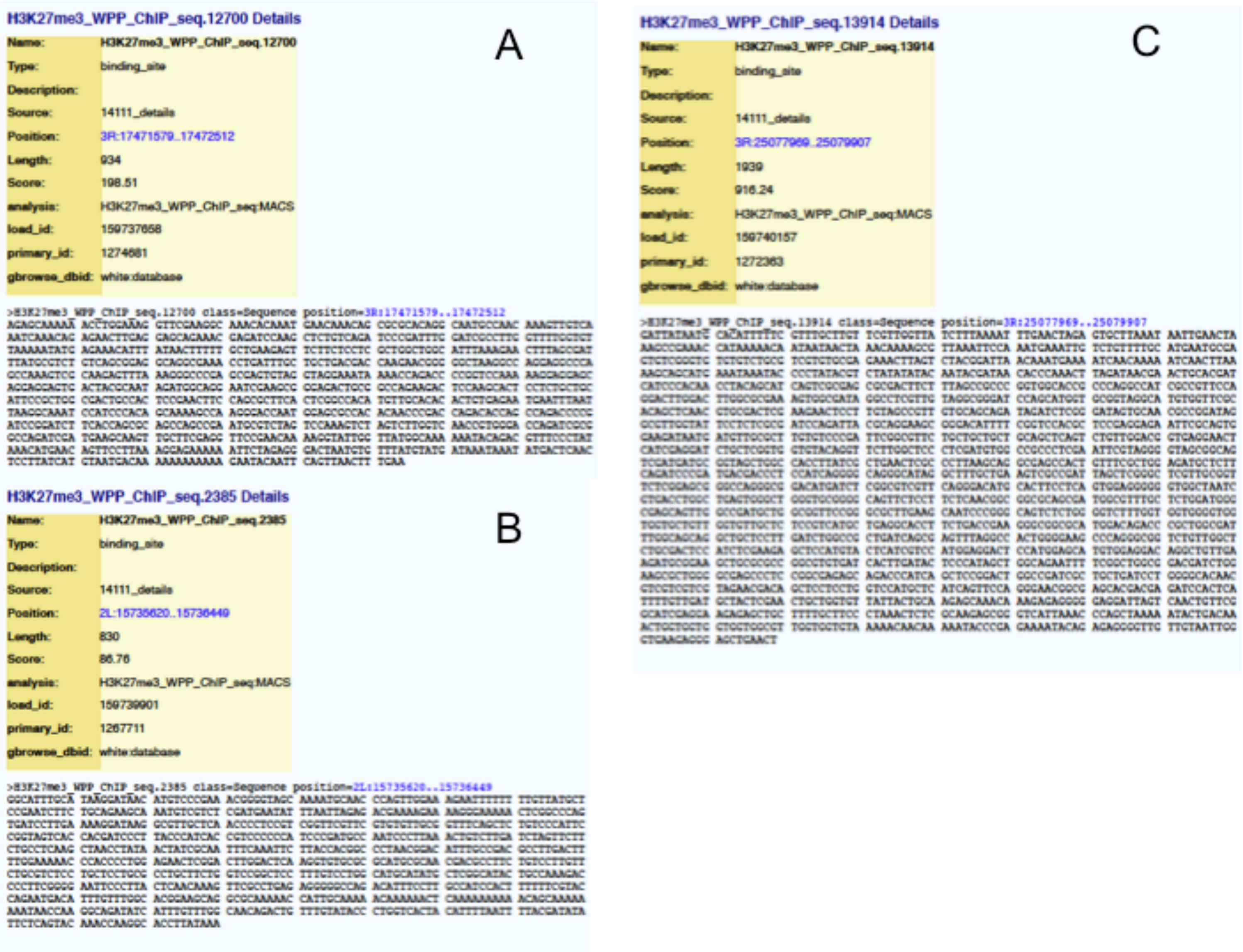

Figure S4 The detailed results of ChIP-Seq analyses showing the enrichment of H3K27me3 in *dE2f1* (**A**, corresponding to the peak in Fig. S1B), *dCycE* (**B**, corresponding to the peak in Fig. S2B) and *stg* (**C**, corresponding to the peak in Suppl. Fig. 3B) loci during the WPP stage. These results are obtained from the ChIP-Seq data sets provided by Dr. Kevin White laboratory from the modENCODE Project (http://modencode.oicr.on.ca/fgb2/gbrowse/fly/).

Table S1 Summary of the genetic analyses using the Exelixis *Df* lines.

Available for download at http://www.g3journal.org/lookup/suppl/doi:10.1534/g3.112.004333/-/DC1.
