# Supplementary material for: In Vivo Regulation of E2F1 by Polycomb Group Genes in Drosophila
Source: G3 (Bethesda). 2012 Dec 1;2(12):1651–60. doi: 10.1534/g3.112.004333 (PMC3516486; doi:10.1534/g3.112.004333)
Supplement: Supporting Information [file supp_2.12.1651_FigureS1.pdf]

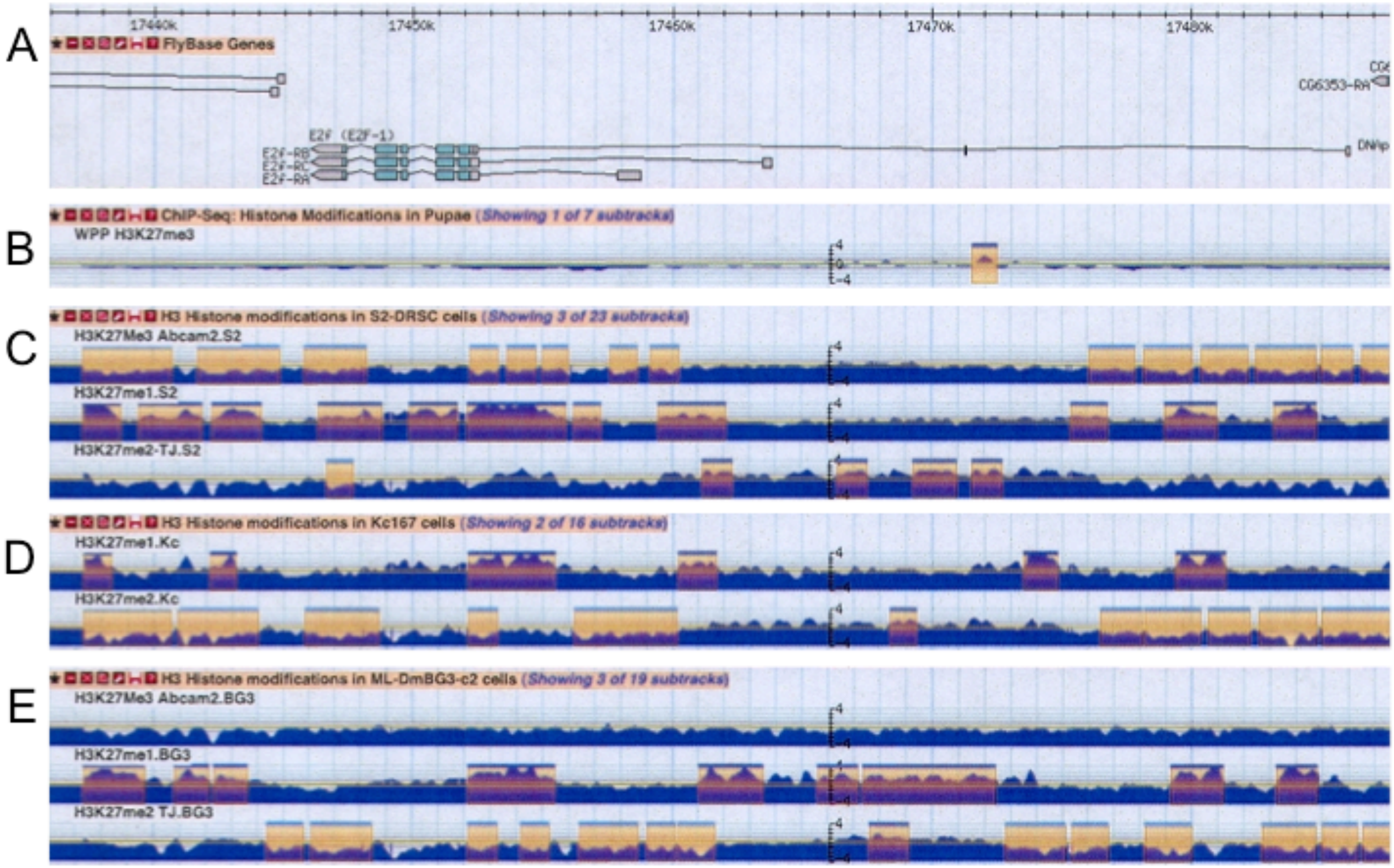

Figure S1 The H3K27me status on the *dE2f1*gene (**A**; Region surveyed: 3R:17444026..17488127) during the white prepupal (WPP) stage (**B**) and in *Drosophila* SL2 (**C**), Kc (**D**) and BG3 (**E**) cells. These results are obtained from the ChIP-chip and ChIP-Seq data sets provided by Drs. Gary Karpen, Vincenzo Pirrotta, and Kevin White laboratories from the modENCODE Project (http://modencode.oicr.on.ca/fgb2/gbrowse/fly/). Note: H3K27me3 is enriched at the *dE2f1* locus during the WPP developmental stage (**B**), and the enrichment of H3K27me1/2 in *dE2f1* locus in all the three cell lines analyzed (**C/D/E**).
