# Supplementary material for: In Vivo Regulation of E2F1 by Polycomb Group Genes in Drosophila
Source: G3 (Bethesda). 2012 Dec 1;2(12):1651–60. doi: 10.1534/g3.112.004333 (PMC3516486; doi:10.1534/g3.112.004333)
Supplement: Supporting Information [file supp_2.12.1651_FigureS3.pdf]

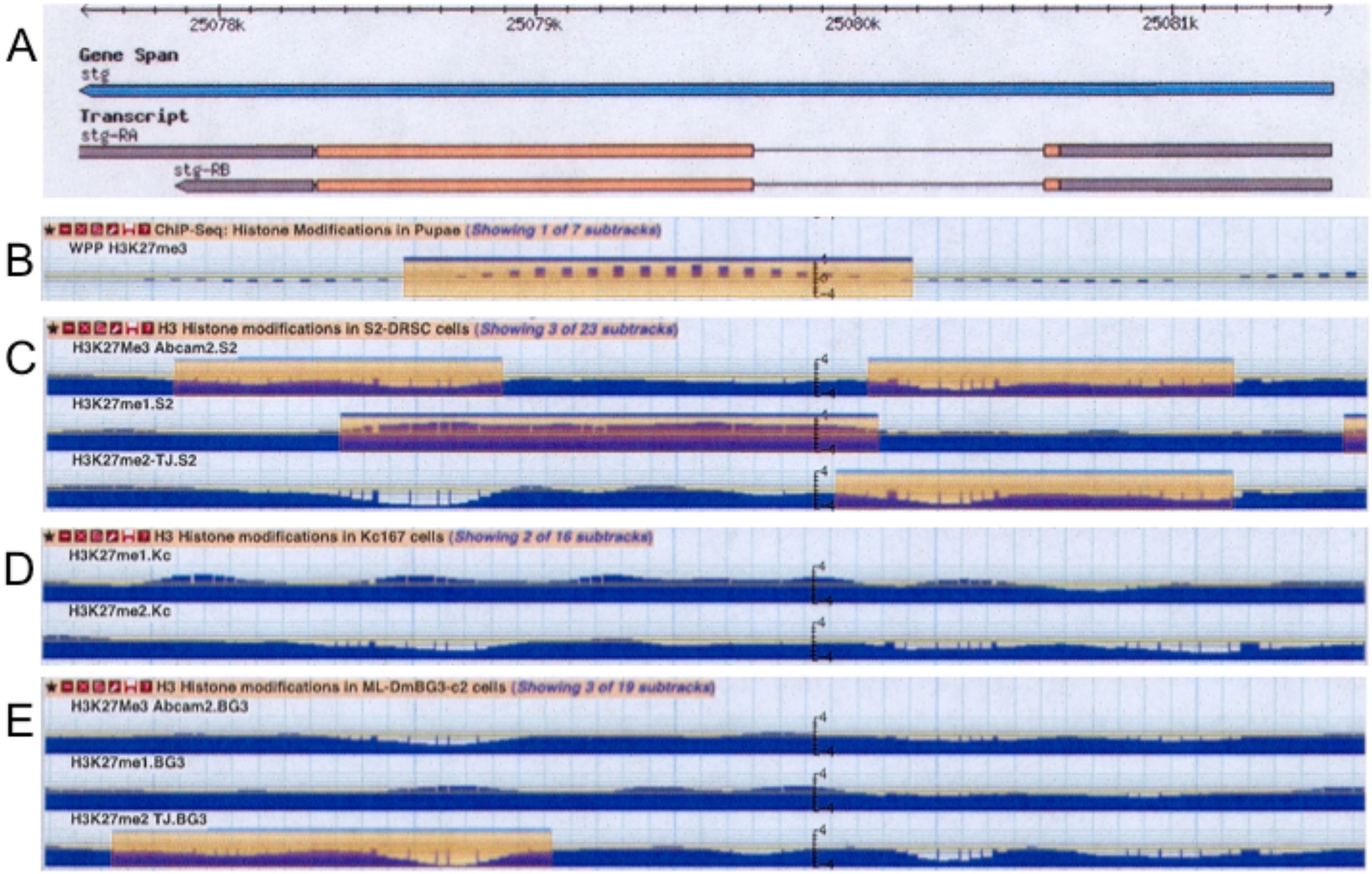

Figure S3 The H3K27me status on the *stg* gene (**A**; Region surveyed: 3R:25064521..25114520) during the WPP stage (**B**) and in *Drosophila* SL2 (**C**), Kc (**D**) and BG3 (**E**) cells. Note the enrichment of H3K27me3 in *stg* locus during the WPP stage (**B**), and the enrichment of H3K27me1 in *stg* locus in SL2 cells (**C**).
