# Supplementary material for: In Vivo Regulation of E2F1 by Polycomb Group Genes in Drosophila
Source: G3 (Bethesda). 2012 Dec 1;2(12):1651–60. doi: 10.1534/g3.112.004333 (PMC3516486; doi:10.1534/g3.112.004333)
Supplement: Supporting Information [file supp_2.12.1651_FigureS4.pdf]

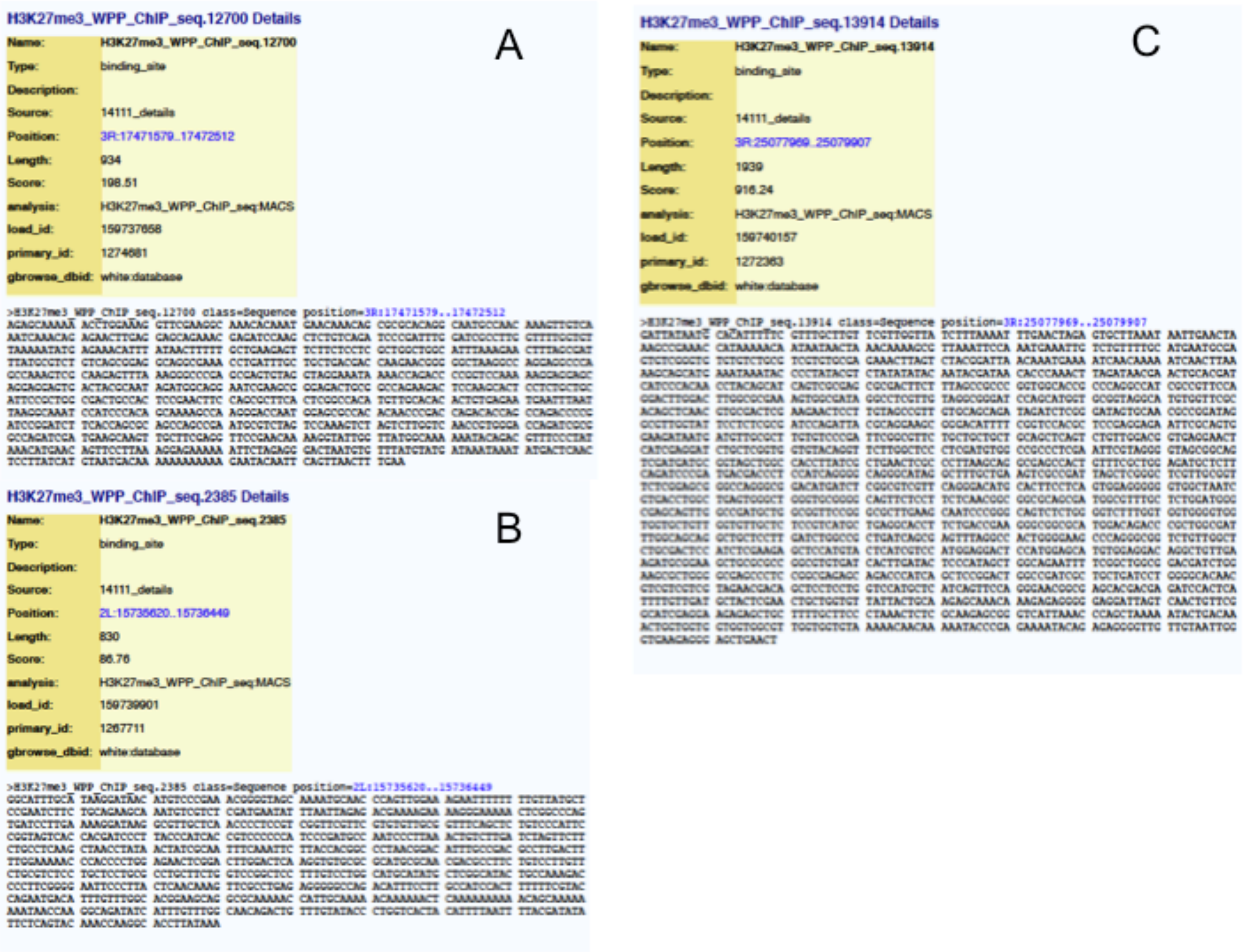

Figure S4 The detailed results of ChIP-Seq analyses showing the enrichment of H3K27me3 in *dE2f1* (**A**, corresponding to the peak in Fig. S1B), *dCycE* (**B**, corresponding to the peak in Fig. S2B) and *stg* (**C**, corresponding to the peak in Suppl. Fig. 3B) loci during the WPP stage. These results are obtained from the ChIP-Seq data sets provided by Dr. Kevin White laboratory from the modENCODE Project (http://modencode.oicr.on.ca/fgb2/gbrowse/fly/).
